# Comparative high-throughput transcriptome sequencing and development of SiESTa, the *Silene *EST annotation database

**DOI:** 10.1186/1471-2164-12-376

**Published:** 2011-07-26

**Authors:** Nicolas Blavet, Delphine Charif, Christine Oger-Desfeux, Gabriel AB Marais, Alex Widmer

**Affiliations:** 1 Institute of Integrative Biology (IBZ), ETH Zurich, Universitaetstrasse 16, Zürich, 8092, Switzerland; 2 Laboratoire de Biométrie et Biologie Evolutive, CNRS UMR 5558, Université Lyon 1, Villeurbanne, F-69622 cedex, France; 3 DTAMB/PRABI, IFR41, Université Lyon 1, Bâtiment Gregor Mendel, Villeurbanne, F-69622 cedex, France

**Keywords:** cDNA library, database, EST, SNP, *Silene*

## Abstract

**Background:**

The genus *Silene *is widely used as a model system for addressing ecological and evolutionary questions in plants, but advances in using the genus as a model system are impeded by the lack of available resources for studying its genome. Massively parallel sequencing cDNA has recently developed into an efficient method for characterizing the transcriptomes of non-model organisms, generating massive amounts of data that enable the study of multiple species in a comparative framework. The sequences generated provide an excellent resource for identifying expressed genes, characterizing functional variation and developing molecular markers, thereby laying the foundations for future studies on gene sequence and gene expression divergence. Here, we report the results of a comparative transcriptome sequencing study of eight individuals representing four *Silene *and one *Dianthus *species as outgroup. All sequences and annotations have been deposited in a newly developed and publicly available database called SiESTa, the *Silene *EST annotation database.

**Results:**

A total of 1,041,122 EST reads were generated in two runs on a Roche GS-FLX 454 pyrosequencing platform. EST reads were analyzed separately for all eight individuals sequenced and were assembled into contigs using TGICL. These were annotated with results from BLASTX searches and Gene Ontology (GO) terms, and thousands of single-nucleotide polymorphisms (SNPs) were characterized. Unassembled reads were kept as singletons and together with the contigs contributed to the unigenes characterized in each individual. The high quality of unigenes is evidenced by the proportion (49%) that have significant hits in similarity searches with the *A. thaliana *proteome. The SiESTa database is accessible at http://www.siesta.ethz.ch.

**Conclusion:**

The sequence collections established in the present study provide an important genomic resource for four *Silene *and one *Dianthus *species and will help to further develop *Silene *as a plant model system. The genes characterized will be useful for future research not only in the species included in the present study, but also in related species for which no genomic resources are yet available. Our results demonstrate the efficiency of massively parallel transcriptome sequencing in a comparative framework as an approach for developing genomic resources in diverse groups of non-model organisms.

## Background

The genus *Silene *(Caryophyllaceae) consists of several hundred species with a mainly holarctic distribution. Because species vary widely in their breeding system, sex determination and ecology, the genus has historically played an important role in genetic and ecological studies dating back to Mendel and Darwin. More recently the genus has emerged as a model system in plant ecology, evolution, genetics and developmental biology [1]. However, a major limitation of using *Silene *as model system is the near absence of genomic information pertaining to the genus. Recently the first EST library was published [2], based on normalized cDNA sequences derived from different reproductive tissues of the dioecious species *Silene latifolia*. In *S. latifolia*, sex is determined by heteromorphic sex chromosomes. As in mammals, *S. latifolia *males are heterogametic (XY) and females are homogametic (XX). In contrast to the evolution of mammalian sex chromosomes which evolved about 150 million years ago (my) [3], the age of *S. latifolia *sex chromosomes has been estimated to be about 10 my [4]. The overwhelming majority of *Silene *species are however not dioecious and lack sex chromosomes. These species are either hermaphroditic or gynodioecious, such as in the case of the widely distributed bladder campion *S. vulgaris*. The relatively recent evolution of sex chromosomes in *S. latifolia *and the availability of closely related species without sex chromosomes, make the genus an ideal target for studying the evolution of sex chromosomes.

The closest relatives of *S. latifolia *are a group of dioecious species, including *S. marizii *and *S. dioica*, with which *S. latifolia *often hybridizes upon secondary contact [5]. The two species occupy different habitats [6] and differ in flower color and odor [7]. As is frequently the case in pairs of closely related plant species where multiple barriers contribute to reproductive isolation [8,9], reproductive isolation between *S. dioica *and *S. latifolia *is incomplete, and the occurrence of gene flow across species boundaries leads to porous genomes [10]. A recent population genomic analysis revealed that neutral processes, introgression and adaptive divergence shape species differences [11]. However, the extent to which genes underlying floral trait or habitat differences contribute to adaptive divergence has never been investigated. A major hindrance to investigate the genetic causes of adaptive divergence is that the *Silene *genome remains largely unexplored. The present study tackles this problem by comparative high-throughput transcriptome sequencing of *Silene latifolia, S. dioica, S. marizii, S. vulgaris *and *Dianthus superbus*. The sequences generated in this study are annotated and publicly available through SiESTa, the *Silene *EST annotation database http://www.siesta.ethz.ch.

*Silene *and *Dianthus *species vary greatly in genome size and have different haploid chromosome numbers (n = 12 and n = 15 respectively). With a haploid genome size of about 2,646 Mbp [12], the *S. latifolia *genome is similar in size to corn (about 2,671 Mbp) [13]. In contrast, the genome size of *S. vulgaris *(1,103 Mbp) [14] is less than half that of *S. latifolia *and some *Dianthus *species have even smaller genomes (613 Mbp) [15]. Thus, genome sizes differ by a factor of two between *Dianthus *and *S. vulgaris *and by a factor of four between *Dianthus *and *S. latifolia*.

To further develop genomic resources for the genus *Silene*, and especially for the dioecious species related to *S. latifolia*, we performed comparative high-throughput transcriptome sequencing using 454 pyrosequencing technology. This method is increasingly used for EST sequencing in both animals [16-18] and plants [19-21]. Advantages over conventional Sanger sequencing based EST projects are the large amount of data generated per run and the fact that cloning is not required as an initial step, factors which substantially reduce the time, labor and cost involved [22,23].

Here we present the results of comparative transcriptome sequencing in seven *Silene *individuals representing four species, and one *Dianthus *outgroup. These species are closely related and include species with and without sex chromosomes, also differing substantially in genome size. A total of 1,041,122 EST reads, totaling 242,341,741 bp, were obtained from two complete 454 pyrosequencing runs and processed and assembled in the SiESTa database. These ESTs provide a unique and novel resource for ecological and evolutionary studies in *Silene *and *Dianthus*.

## Results and Discussion

### SiESTa database characteristics

454 pyrosequencing of eight individual cDNA libraries derived from one *Silene latifolia *male (SlM) and two females (SlF, SlFf), one *S. dioica *male (SdM) and female (SdF), one *S. marizii *male (SmM), and one individual of the each of the hermaphroditic species *S. vulgaris *(SvH) and *Dianthus superbus *(Ds) lead to a total of 1,041,122 EST reads. The number of nucleotides sequenced per library varied between 25 million and 46 million in SlFf and Ds respectively (Table 1). In contrast to studies using normalized libraries [17,19,21,24], we used non-normalized libraries with the advantage of searches not identifying weakly expressed genes and a reduced chance of finding alternative splicing variants [25]. However, these factors may negatively impact upon the ability to build contigs. Our reads were assembled into 93,627 contigs (38,256,084 bp) and 309,074 singletons (69,524,702 bp), with an overall total of 402,701 unigenes (107,780,786 bp) that were deposited in a newly developed database called SiESTa (*Silene *EST annotations) (Table 1). All reads may be accessed under the accession number ERP000371 in the NCBI Sequence Read Archive and all contigs are available in Genbank Transcriptome Shotgun Assembly (TSA) under the accession numbers JL382689 - JL473671. The unigenes were sorted into eight individual libraries with an average size of 130,140 ESTs and 11,703 contigs per library. Two super-libraries, supSL and supSD, containing the sequences of *S. latifolia *and *S. dioica *individuals, respectively, were also created. Their sizes are 129,456 and 129,252 superunigenes respectively.

**Table 1:** SiESTa sequence content

	Library									
	
	SlM	SlF	SlFf	SdM	SdF	SmM	SvH	Ds	supSL	supSD
# ESTs	119	136	110	113	115	127	123	198	347	228
Nucleotides (Mbp)	28	32	25	27	27	29	29	46	85	54
# Unigenes	61	40	49	71	69	51	32	30	129	129
% Contigs	17	34	17	15	17	28	36	43	24	18
% ESTs in contigs	57	81	63	47	50	71	83	91	72	54
Avg. EST length (bp)	235	232	225	235	233	230	234	232	230	233
Avg. contig length (bp)	403	430	413	395	385	392	401	463	422	396

(#) Units in thousands of sequences.

As reported elsewhere in recent studies [18,21,24] short EST reads from 454 sequencing runs may be assembled and annotated to effectively characterize the gene space of non-model organisms. Average read length in our study was 232 bp, close to the lengths obtained in other recent studies that used the GS-FLX platform for sequencing [17,18,21], but substantially longer than early studies that used the GS-20 platform where read lengths were 100 to 110 bp [16,19,21]. Between 47% and 91% of EST reads were assembled into contigs (for SdM and Ds respectively), while the remainder were kept as singletons (Table 1). Similar percentages of reads assembled into contigs were found in other studies, ranging from 40% to 48% [19,25] to 88% and 90% [16-18,21] in both plants and animals. The frequency distribution of ESTs per unigene showed a hyperbolic distribution (Additional file 1), with a single EST read available for most unigenes (singletons), whereas only a small proportion of unigenes include a large number of EST reads. Given that our libraries were not normalized, one can use the number of ESTs per unigene as an estimate of expression level [26]. This implies that the unigenes composed of many EST reads are highly expressed. An analysis of the ten most strongly expressed genes (i.e. the unigenes with the highest numbers of EST reads) in each library revealed that these correspond to only fifteen different genes (Additional file 2). Of these, two were found in more than four out of the eight libraries analyzed. Our results indicate that one of these genes codes for an alpha-tubulin homologue of *Arabidopsis thaliana *(present in SlM, SlFf, SdM, SdF, SvH, Ds) and the second for a homologue of a predicted ORF in *Pinus koraiensis *(present in SlM, SdM, SdF, Ds) (Additional file 2). Most of these genes are housekeeping genes that are known to be highly expressed [27-30].

GO annotations revealed that a large number of contigs had a term assigned to them. Of the 93,048 contigs tested (from supSL, supSD, SmM, SvH and Ds), 46,217 were annotated with a GO term. The large number of GO terms annotated in the libraries (53%) further confirms the quality of the contigs of our database.

**Figure 1:**
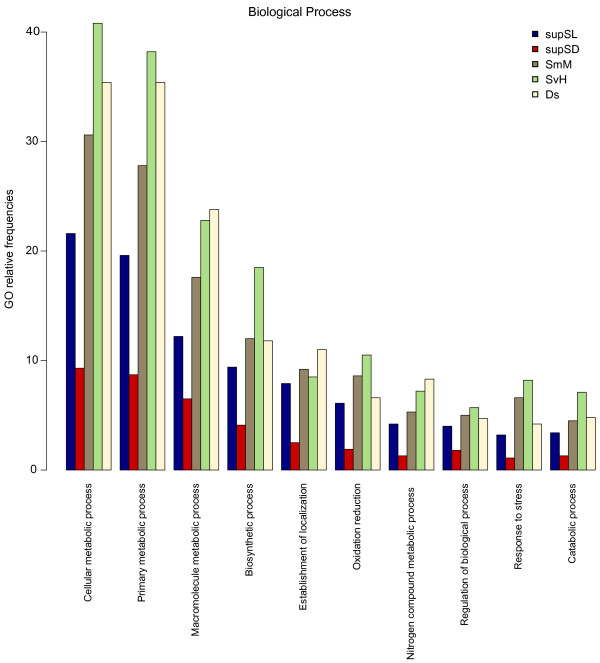
**Relative frequencies of the most represented Biological Process GO sub-classes across libraries**. Figure 1 shows the ten most frequent biological process GO terms at level 3 in the five species *Silene latifolia, S. dioica, S. marizii, S. vulgaris *and *Dianthus superbus*.

A comparison of the ten most represented GO annotations reveals substantial homogeneity in the composition of our libraries (Figure 1 and Additional file 3).

In addition, the analysis of the ten most represented gene groups, based on the most expressed GO Slim for plants [31], confirmed the homogeneity of gene expression in the buds of the different species studied (Table 2). Not surprisingly, genes involved in cellular component organization translation and transcription are highly expressed in all our individuals.

**Table 2:** Expression differences among all eight libraries for the ten most frequently represented GO Slim terms

GO Slim term	Expression percentage							
	
	SlM	SlF	SlFf	SdM	SdF	SmM	SvH	Ds
Response to stress	2.3%	8.1%	2.3%	1.5%	2.8%	6.6%	8.2%	4.2%
Cellular component organization	2.6%	2.9%	2.0%	1.2%	2.0%	3.7%	3.5%	7.5%
Translation	1.3%	7.3%	1.8%	1.0%	4.7%	2.2%	3.4%	2.5%
Photosynthesis	2.2%	6.2%	2.3%	0.8%	2.7%	4.6%	3.8%	0.4%
Kinase activity	2.8%	2.7%	2.5%	0.8%	1.1%	3.2%	3.2%	4.6%
Cell communication	2.3%	2.2%	2.7%	1.2%	1.7%	2.1%	1.9%	2.2%
Signal transduction	2.2%	2.2%	2.6%	1.1%	1.6%	2.1%	1.9%	2.1%
Response to abiotic stimulus	1.0%	3.5%	1.2%	0.5%	1.2%	2.5%	3.1%	2.2%
Transcription	1.2%	1.8%	1.1%	0.8%	1.8%	1.3%	1.4%	1.4%
Response to biotic stimulus	0.4%	2.1%	0.4%	0.3%	0.5%	1.2%	1.4%	0.2%

(Biological processes and Molecular functions). For each library, the expression percentage is calculated as the number of reads included in contigs matching a term divided by the total number of reads included in all contigs. Terms are sorted by the total expression common to all libraries in descending order.

Ninety-nine percent of the unigenes have an ORF predicted by prot4EST (Table 3). About 45% of the predictions are based on BLAST similarities, 28% are predicted by ESTScan and the remaining 27% correspond to the longest reading frames of the sequences.

**Table 3:** Prot4EST ORF prediction results

Library	Predicted ORFs				
	
	Similarity	ESTScan	Longest ORF	Average length	Total
supSL	39%	31%	31%	208	129251
supSD	29%	36%	35%	193	129154
SmM	36%	28%	35%	205	50798
SvH	60%	24%	16%	245	32131
Ds	59%	23%	18%	259	29668

ORF prediction based on similarity with BLAST results, ESTScan prediction and longest reading frame.

The *Silene *genome is known to include a large number of repeated elements [32-34] and we had to filter out such elements because they contribute to assembly problems. On average, 23,000 reads per library matched repeated elements (data not shown). Numerous repeated elements have recently been identified in *S. latifolia *[32], which make easier contig construction even a large diversity of elements still remains to be characterized.

Our newly developed EST resources for *Silene *and *Dianthus*, with 130,140 ESTs on average, are comparable to the resources available for *Helianthus annuus *(133,684 ESTs) and for *Populus trichocarpa *(89,943 ESTs) [NCBI EST database of October 1, 2010].

### The SiESTa database

The newly developed SiESTa database provides several tools that facilitate data and information extraction. The first tool is the unigene search engine (Unisearch), which allows to enter a list of unigene or superunigene names. From these, the user directly obtains the link to the sequence annotations and can download all sequences in fasta format. The second tab called "Libraries" allows users to navigate the database. Information about the different libraries, including species identity and tissue used, sex of the individual and the total number of unigenes/superunigenes in the library are presented in a table. By selecting the link on the unigene number, the user may download the complete set of unigenes from each library. The link attached to the library name enables the user to access the unigene table which lists unigenes, their lengths, the number of ESTs per unigene and the best hit with Uniprot. Selecting any unigene provides access to the unigene sequence, a picture of the EST alignment that is linked to the alignment in fasta format and a table with the five best hits with the *A. thaliana *proteome and Uniprot. In the case of superunigenes, additional information is available, including ORF prediction and the list of unigenes that are part of the superunigene. The third tool, "Query", allows users to search for genes using their annotations. The fourth tab provides a link to a Gene Onthology formatted browser interface from which it is possible to obtain GO annotation for contigs of each species included in SiESTa. The fifth tool is a BLAST search engine that allows users to search for nucleic or protein sequence homology within the eight SiESTa libraries using BLASTN, TBLASTX or TBLASTN searches. The sixth and seventh tabs "Tools" and "FAQ" provide all this information on the web-site.

### Homology with plant model species

In order to annotate and evaluate the quality of our reads and of our assemblies, we performed BLASTX searches to align both contigs and singletons from each library with *A. thaliana, Vitis vinifera *and *Populus trichocarpa *proteomes and Uniprot (Tables 4). On average, 49% of the contigs and 27% of the singletons had a significant hit to the *A. thaliana *proteome. We evaluated the redundancy of the hits and found that on average 32% and 18% of contigs and singletons respectively match strictly different *A. thaliana *protein sequence. These non-redundant protein sequences (noted 'unique' in Table 4) revealed that some of our unigenes could come from distinct regions of the same gene. Compared to the proportions of hits with *A. thaliana*, we noticed an increase of the average percentage of matches for both contigs and singletons respectively, with *V. vinifera *(+0.2% and +1.9%), *P. trichocarpa *(+1.9% and +5.7%) and with Uniprot (+8.8% and +14.7%). Nevertheless, even though most of the *Silene *genes have a match with the three model species, across all libraries, an average of 62, 87 and 189 hits are exclusive to the proteomes of *A. thaliana, V. vinifera *or *P. trichocarpa*, respectively (Table 5). Such differences among the investigated proteomes might suggest that *P. trichocarpa *is more closely related to *Silene *than *A. thaliana *and *V. vinifera*. However, the phylogeny of angiosperms compiled by Bremer and coworkers [35] reveals that *Silene *(Caryophyllales) is phylogenetically equally distant from *Vitis *(Vitales), *Populus *(Malpighiales) and *Arabidopsis *(Brassicales). The causes of these observed differences are currently unknown, but a possible explanation may be differential gene loss during the evolution of these plant lineages as observed in other plants [36,37].

**Table 4:** BLASTX hits of contigs and singletons in the eight individual libraries with different proteomes

Library	Contigs							
	
	*A. thaliana*		*V. vinifera*		*P. trichocarpa*		Uniprot	
	
	%hit	% unique	%hit	% unique	%hit	% unique	%hit	% unique
SlM	31%	21%	31%	20%	33%	23%	41%	33%
SlF	76%	49%	76%	46%	78%	53%	78%	66%
SlFf	35%	24%	35%	24%	36%	26%	56%	47%
SdM	18%	13%	19%	13%	22%	14%	31%	22%
SdF	31%	22%	31%	21%	32%	24%	49%	43%
SmM	55%	35%	54%	33%	56%	39%	57%	48%
SvH	74%	48%	74%	45%	76%	52%	76%	64%
Ds	73%	47%	73%	43%	74%	51%	75%	63%

								

Library	**Singletons**							
	
	***A. thaliana***		***V. vinifera***		*** P. trichocarpa***		**Uniprot**	
	
	%hit	% unique	%hit	% unique	%hit	% unique	%hit	% unique
SlM	14%	9%	16%	9%	21%	10%	32%	19%
SlF	42%	27%	44%	26%	46%	31%	47%	38%
SlFf	17%	11%	18%	11%	22%	12%	45%	30%
SdM	10%	6%	13%	6%	21%	7%	32%	14%
SdF	16%	10%	18%	10%	22%	11%	39%	25%
SmM	26%	17%	28%	17%	30%	20%	32%	25%
SvH	44%	30%	47%	29%	50%	33%	55%	41%
Ds	46%	33%	46%	32%	48%	36%	49%	42%

Table 4 shows the number of hits for both contigs and singletons. Non-redundant accessions are recorded in the '% unique' column. A cut-off E-value of 1E-4 was used for each database.

**Table 5:** *Silene *contigs with hits that are exclusive to the *A. thaliana, V. vinifera*, and *P. trichocarpa *proteomes

Library	# hits in At, Vv, Pt	# hits At	# hits Vv	# hits Pt
SlM	2886	74	78	220
SlF	9887	49	102	152
SlFf	2732	69	104	105
SdM	1758	32	77	391
SdF	3339	99	70	212
SmM	7418	66	107	187
SvH	8270	44	71	130
Ds	8885	64	93	120
Mean	5646	62	87	189

In the second column are numbers of contigs with hits occurring in all three species; the following columns give the numbers of contigs with hits exclusively to *A. thaliana *(At) (3^rd ^column) (these sequences do not have significant matches with either *V. vinifera *or *P. trichocarpa*), *V. vinifera *(Vv) (4^th ^column) and *P. trichocarpa *(Pt) (5^th ^column).

### Contigs lacking known homologs

**Figure 2:**
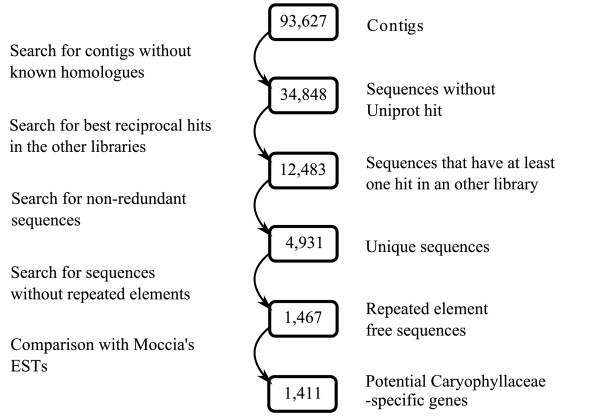
**Identification of potential Caryophyllaceae-specific genes**. The first step identifies sequences without known homologues in reference species; the second and the third steps select sequences that are found in at least two SiESTa libraries. Sequences that partially match repeated elements are removed. In our final step we compared the remaining sequences with the *Silene *EST library of Moccia et al. [2] to identify potential Caryophyllaceae-specific genes.

For 34,848 contigs out of the 93,627 contigs assembled in the present study, homologous genes could not be identified through BLAST searches against several databases (Additional file 4). Of these 34,848 contigs, 22,365 were found only in a single library, whereas 12,483 contigs correspond to sequences found in more than one library. After removing redundancies, 4,931 unigenes remained that had no significant hit in BLASTX searches against Uniprot and were found in at least two libraries. A substantial proportion of these unigenes (69%) had similarities with additional repeated elements identified from *S. latifolia *(J. Macas, unpublished results) and were removed. The remaining 1,467 contigs were compared with the EST library reported by Moccia et al. [2], and 14% of these contigs had a significant hit. For some of the corresponding ESTs of Moccia et al. [2] there is a significant hit with Uniprot, most likely because these sequences were longer and contained coding sequences, and we were able to infer homology for 56 contigs. After removing one further transposon homologue and 40 sequences consisting of UTR regions, only 15 sequences had a good homology with gene coding regions, but 7 of them had undetermined functions. Of the remaining contigs, 1,411 sequences looked like potential Caryophyllaceae-specific genes (Figure 2). Yang and coworkers [38] recently investigated species-specific genes in the *A. thaliana, P. trichocarpa *and *Oryza sativa *proteomes. Inter-proteome comparisons revealed that 165 of 26,784 proteins (0.6%) are exclusive to *A. thaliana*, as these proteins have no homologue in either *P. trichocarpa *or *O. sativa *and also in *Carica, Glycine, Medicago, Sorghum, Vitis *and *Zea *(similar results are indicated in *P. trichocarpa and O. sativa*). Similarly, we searched our libraries for genes that are specific for *Silene or Dianthus*. We have identified 1,411 sequences from our studied species that may correspond to Caryophyllaceae-specific genes. These sequences represent about 1.5% of all contigs. The proportions of species-specific proteins identified by Yang et al. [38] in *A. thaliana, P. trichocarpa *and *O. sativa *are 0.6%, 0.2% and 1.1% respectively. Our estimate is also less than 2%, but we do not have a sequenced genome available, and consequently, some genes are certainly missing in the calculation and some may have been counted more than once. Possible biases introduced in our estimates include that 1) we used contigs built from cDNA sequences. These are different from full-length protein sequences because they are oftentimes only fragments of coding sequences and it is possible that different contigs contain non-overlapping regions of the same gene as revealed by Table 4. 2) Singletons were not included in this analysis because their quantity prevented computation. 3) The lack of well-annotated genome sequences of species closely related to *Silene *reduced chances to find more homologous sequences. 4) Our EST libraries were non-normalized, and it is thus possible that further Caryophyllaceae-specific genes were missed because they were not sufficiently expressed to be represented in our database. Points 2 and 4 might increase the proportion of Caryophyllaceae-specific genes while points 1 and 3 might decrease it. Further studies will reveal whether these sequences are indeed specific to Silene and what their functions are. For this purpose, our SiESTa database provides a valuable resource.

### SNP detection, validation and heterozygosity estimates

QualitySNP predicted between 4,500 and 12,000 polymorphic sites in our eight libraries, the results of SNP analysis for each individual library being presented in Table 6. There are on average 31 SNPs per 10,000 bp of expressed sequences in *Silene *and *Dianthus *and most SNPs are substitutions (78.6%).

**Table 6:** Library SNP content

Library	SlM	SlF	SlFf	SdM	SdF	SmM	SvH	Ds
Contigs*	2909	5486	2287	2993	2912	4982	5028	6094
Contigs with SNPs	1221	1517	976	1333	1078	1709	1619	2513
SNPs	6648	6308	5576	6307	4653	7361	7381	12282
Substitutions	4847	5165	3873	5356	3516	5681	6402	9927
% Transitions/transversions	52/48	61/39	47/53	63/37	56/44	57/43	60/40	61/39
% heterozygous positions	0.39	0.19	0.43	0.38	0.28	0.26	0.27	0.32

* Only contigs assembled from at least 4 reads were considered. The total length of these contigs was used to calculate the percentage of heterozygous positions. All SNPs that are not due to substitutions are indels.

Of the 48 polymorphic positions predicted by qualitySNP that were selected for validation, 32 (67%) of SNPs were confirmed by Sanger sequencing of PCR products. Polymorphic positions that are not associated with single-nucleotide repeats were selected for validation, because pyrosequencing is known to experience difficulties in sequencing these regions [39]. We observed that such regions often induce incorrect predictions of SNPs by qualitySNP. From our SNP data we cannot directly estimate nucleotide diversity, because our SNP estimates are based on reads from single individuals. However, the detected polymorphisms allow estimating heterozygosity in the different species. Polymorphism varies between 19 and 43 SNPs per 10 kb of expressed sequences for SlF and SlFf respectively (Table 6). Similar values were reported in maize [20], with between 33 and 47 SNPs per 10 kb, in *Oryza sativa *[40], with around 30 SNPs per 10 kb, and in *A. thaliana *[41], with around 40 SNPs per 10 kb. By analyzing 27 genes in *Silene latifolia*, a recent study estimated a polymorphism rate of about 551 SNPs per 10 kb [42], which is ten times higher than in other plants. The origin and the large number of individuals sampled in that study is probably the reason for these high estimates. Our results suggested that there is no difference in the proportion of heterozygous positions between the dioecious species and the gynodioecious species in the genus *Silene *(mean of 0.32 and 0.3 respectively). However, a lower level of heterozygosity was detected in the *S. latifolia *female library SlF (0.19) compared to other libraries. SIF belongs to an inbred line, which explains the low polymorphism exhibited by this individual. On the contrary, an increase of polymorphism was detected in the F1 individual SlFf, which was obtained by crossing the two other *S. latifolia *plants, SlM and SlF. Polymorphisms detected in this individual provide valuable markers for the development of a linkage map for *S. latifolia *and its sex chromosomes.

## Conclusions

The high quality EST database SiESTa provides valuable resources for molecular ecologists studying Caryophyllaceae, particularly for the genus *Silene*. It provides the necessary molecular resources to develop microsatellite and SNP markers for linkage mapping and population genetic analyses, provides access to candidate genes for specific traits, such as heavy metal tolerance or flower color variation, and enables identification of × and Y-linked gene copies. Moreover this online database http://www.siesta.ethz.ch provides access to sequences and annotations of four *Silene *and one *Dianthus *species lacking fully-sequenced genomes. The two 454 sequencing runs described in this study generated more than one million sequencing reads, allowing for the identification of about 74,000 genes and about 56,000 SNPs. We hope that the availability of these resources will encourage further investigations into the genomics and evolutionary biology of *Silene *and related species.

## Methods

### RNA extraction & cDNA sequencing

We extracted RNA from one flower bud belonging to eight individuals of five closely related species; three dioecious species: *Silene latifolia, S. dioica *and *S. marizii *and two gynodioecious species: *S. vulgaris *and *Dianthus superbus*. For *S. latifolia *and *S. dioica*, both sexes were included in this study, whereas for *S. marizii*, only a male individual was used. For the two gynodioecious species, we used flowers from hermaphrodite individuals.

Flower buds prior to anthesis were collected from plants grown in a greenhouse under long day conditions at the ETH Zurich and were immediately frozen in liquid nitrogen. Flower buds of *Dianthus superbus *were collected in the field (Davos, Switzerland) and immediately placed in RNALater (Ambion) and stored at room temperature for three days. Total RNA was isolated using TriFast (PeqLab), stored in liquid nitrogen, and sent to GATC Biotech (Konstanz, Germany) for library construction. cDNA was prepared using the SMART™ PCR cDNA Synthesis Kit (Clontech), concatenated by ligation, nebulized, tagged and sequenced using the GS FLX protocol (Roche). Two tagged libraries were combined in half a picotiter plate for sequencing.

### EST processing

All sequences were generated in two complete runs on a Roche GS-FLX 454 pyrosequencing machine and eight fasta files containing trimmed reads were extracted from the sff files. Short reads (< 50 nt), as well as reads derived from mitochondria and plastids were removed using SeqClean [43]. Repeated elements were then removed using RepeatMasker [44] with a Viridiplantae database compiled in RepBase (01/08/2008 version) [45] to which we added the *Silene latifolia *- specific repeated elements identified by Cermak and coworkers [32]. EST reads were then clustered and contigs built using TGICL [46] with the default parameters (95% of identity and 40 bp minimum for sequence overlap). In addition to the resulting contigs, the remaining singletons (unique EST reads) were then added to the database as unigenes.

We constructed separate EST libraries from all eight individuals used in this project: a *Silene latifolia *male (SlM), two *S. latifolia *females, which are "mother" and "daughter" (respectively SlF and SlFf), one male and female each of *S. dioica *(SdM and SdF, respectively), a *S. marizii *male (SmM), as well as one individual each of *S. vulgaris *(SvH) and *Dianthus superbus *(Ds). Additionally, two super-libraries were constructed that combine sequences from the three *S. latifolia *(called supSL) and the two *S. dioica *individuals (supSD), respectively. Due to large demand placed on CPU use, ESTs from chloroplast and mitochondrial genes were removed from the assembly process for supSL, thereby reducing the number of reads used from 365,089 to 347,047.

### Unigene annotation

Similarity searches were carried out in two steps, the first of which involved BLAST similarity searches [47] of the contig sequences versus Uniprot (UniProt Rel. 13 = SWISS-PROT 55 + TrEMBL 38, 29 April 2008) and added the annotation results to the database. Because of the large number of contigs being searched for similarities, we used PC clusters at the French National Institute of Nuclear Physics and Physics of Particles located in Lyon (IN2P3). In our second step, BLASTX searches against the *Arabidopsis thaliana *proteome were then performed and the results included in the database. In both steps, the E-value cut-off used was 1E-04 and the five best hits were included in the SiESTa database.

Prot4EST [48] was then used to predict open reading frames (ORFs) using the following criteria: 1) if a unigene had a significant BLAST hit with Uniprot, the ORF from the best hit was used as template for ORF prediction; 2) if the unigene had no BLAST hit, ESTScan [49] predicted peptides that were used to predict ORFs. We used *Arabidopsis thaliana *as 'training model' for ESTScan prediction (codon usage matrix from May 2009 [50]); 3) if EST Scan failed to predict any peptides, the longest ORF from the 6-frame was retained. In addition to prot4EST predictions, we retained all ORFs that were at least 180 bp long when they were in a different reading frame than the prediction done at step 2) and 3). We ran prot4EST on individual libraries Ds, SmM and SvH, and on the super-libraries supSL and supSD. Predicted ORFs were added to the database. Gene ontology (GO) annotation was carried out using Blast2GO [51]. In the mapping step, a pool of candidate GO terms was obtained for each unigene by retrieving GO terms associated with the 20 first BLAST hits (BLASTX against NCBI nr: E-value cut-off: 1e-3; HSP coverage percentage: 0.33). In the annotation step, reliable GO terms were then selected from the pool of candidate GO terms by applying the core annotation function of Blast2GO. Default parameters were used (GO weights: 5; score threshold: 55; Evidence code weights: default). In order to complete the functional annotation (based on BLAST) with protein domain information, InterproScan [52] was run (based on the longest unigene's ORF) and GO terms associated with protein domains were merged with the GO terms kept at the annotation step.

We used the tools provided by the GO consortium to build our own GO database dedicated to our species by loading the 'unigene products - GO' association files found with Blast2GO [http://www.geneontology.org/godatabase/archive/full/2009-03-01/]. To search and browse the gene ontology and visualize the gene products associated with a particular GO term, we implemented an instance of the Amigo browser [http://www.geneontology.org/GO.tools.browsers.shtml].

### Homology investigation

Annotation of the unigenes led to the identification of two major groups of unigenes: the first one with matches to Uniprot and the second without matches. We used the contigs in the first group to estimate the proportion of homologous genes shared with the plant model species *Arabidopsis thaliana, Vitis vinifera *and *Populus trichocarpa*.

We then used unigenes of the second group, i.e. unigenes for which no hits with Uniprot were obtained, to assess whether potentially new, Caryophyllaceae-specific genes could be found. To avoid spurious results, we removed all unigenes in this group that were found only in a single individual. To do so, we performed pairwise BLASTN searches between libraries and removed all sequences without hits in other libraries. From the sequences with hits in other libraries, we kept only one sequence for further analysis. Finally, using the database recently developed by Macas and coworkers (unpublished results) of newly identified *S. latifolia *repeated elements, we tested whether the remaining contigs contained repeated elements that were not removed by RepeatMasker. When more than 20% of the contig length resulted from repeated elements, contigs were discarded. We then compared the remaining sequences with the EST library developed by Moccia and coworkers [2]. This EST library was established by standard Sanger sequencing of a normalized cDNA library. It contains only 3105 unigenes, but these are on average longer than our 454-based unigenes. Moreover, these unigenes were used in the construction of a custom cDNA microarray that has been used in expression analyses by Aria Minder (unpublished results). This comparison firstly allowed us to identify homology that we missed due to the commutative property of homologies and secondly, to assess the proportion of genes that lack annotation which are expressed in *S. latifolia*.

### SNP detection, validation and heterozygosity estimation

SNPs were identified using qualitySNP [53], a haplotype-based SNP finder that groups sequences sharing the same nucleotides at each polymorphic site using the resulting clusters defined with CAP3 [54] and predicts if the SNP position is supported. We used the CAP3 clusters build during TGICL assembly. We ran qualitySNP with the default parameters on the contigs of each individual library. The software searched for polymorphisms in contigs formed by at least four ESTs and identified all potential SNPs that occurred at least twice. Tips of each sequence (30 bp in 5' end and 20% of sequence length in 3' end) were set as low quality (LQ) regions and the rest as high quality regions following the method used by Tang and coworkers [53]. Only high quality SNPs with a minimum score of two were retained. Since we did not search for polymorphisms common to individuals but rather within individuals, SNPs identified by qualitySNP were used to estimate heterozygosity within each individual.

In order to confirm their quality, substitutional SNPs identified by qualitySNP were selected for validation. Specifically, we selected unigenes present in both libraries, SlM and SlFf, that contained SNPs (i.e. heterozygous positions in either SlM or SlFf). SNP Primers were designed using Primer3 [55] to be located at least 40 bp from the SNP and PCR amplify fragments of at least 200 bp in length. Seventeen primer pairs were designed in order to validate 48 SNPs.

The SiESTa database may be accessed using the login and password below at http://www.siesta.ethz.ch

*login: *5!LeN3

*password: *4cent5ante4

## Authors' contributions

NB collected and analyzed data and drafted the manuscript. DC designed the database, analyzed the data and assisted in writing. CO participated in data capture. GABM participated in coordinating the study and assisted with data analysis, interpretation and preparation of the manuscript. AW conceived and coordinated the study and assisted with drafting the manuscript. All authors read and approved the final manuscript.

## Supplementary Material

Additional file 1 **Distribution of ESTs per unigene**. Distribution of EST reads per unigene in the SlF library. The x-axis represents EST reads per unigene and the y-axis the number of unigenes. Click here for file

Additional file 2 **Highly expressed genes**. Fifteen genes were identified among the ten most strongly expressed genes in each library. The two first genes coding for homologues of alpha-tubulin and an unknown gene in *Pinus koraiensis *are present in at least four of our libraries. 'Presence' indicates the number of libraries in which a given gene was found among the ten most strongly expressed genes. E-value cut-off 1E-4. Click here for file

Additional file 3 **Relative frequencies of the most represented Molecular Function GO sub-classes across libraries**. Additional Figure 2 shows the 10 most frequent molecular function GO terms at level 3 in the five species *Silene latifolia, S. dioica, S. marizii, S. vulgaris *and *Dianthus superbus*. Click here for file

Additional file 4 **BLAST hits of unigenes in the eight individual libraries with different databases**. BLAST hits of unigenes in the eight individual libraries with the following databases (E-value cut-off 1E-4): AT = *Arabidopsis thaliana*, VV = *Vitis vinifera*, PT = *Populus trichocarpa*, SL = *Silene latifolia*. Protein sequences were downloaded from: http://plants.ensembl.org/info/data/ftp/index.html: AT proteome = TAIR10.pep 07.02.2011, VV proteome = IGGP12x.pep 07.02.2011, PT proteome = JGI2.0.pep 07.02.2011. AT EST = AGI_release_15, VV EST = VVGI_release_7, PT EST = PPLGI_release_5. SL mtDNA is *S. latifolia *mtDNA described in (Sloan et al., 2010). Uniprot release 07.2010. *A. thaliana *mtDNA, cpDNA, exon, intron, intergenic, 3' UTR and 5' UTR come from TAIR10. Click here for file
