# Supplementary figures and images for: Comparative high-throughput transcriptome sequencing and development of SiESTa, the Silene EST annotation database
Source: BMC Genomics. 2011 Jul 26;12:376. doi: 10.1186/1471-2164-12-376 (PMC3157477; doi:10.1186/1471-2164-12-376)

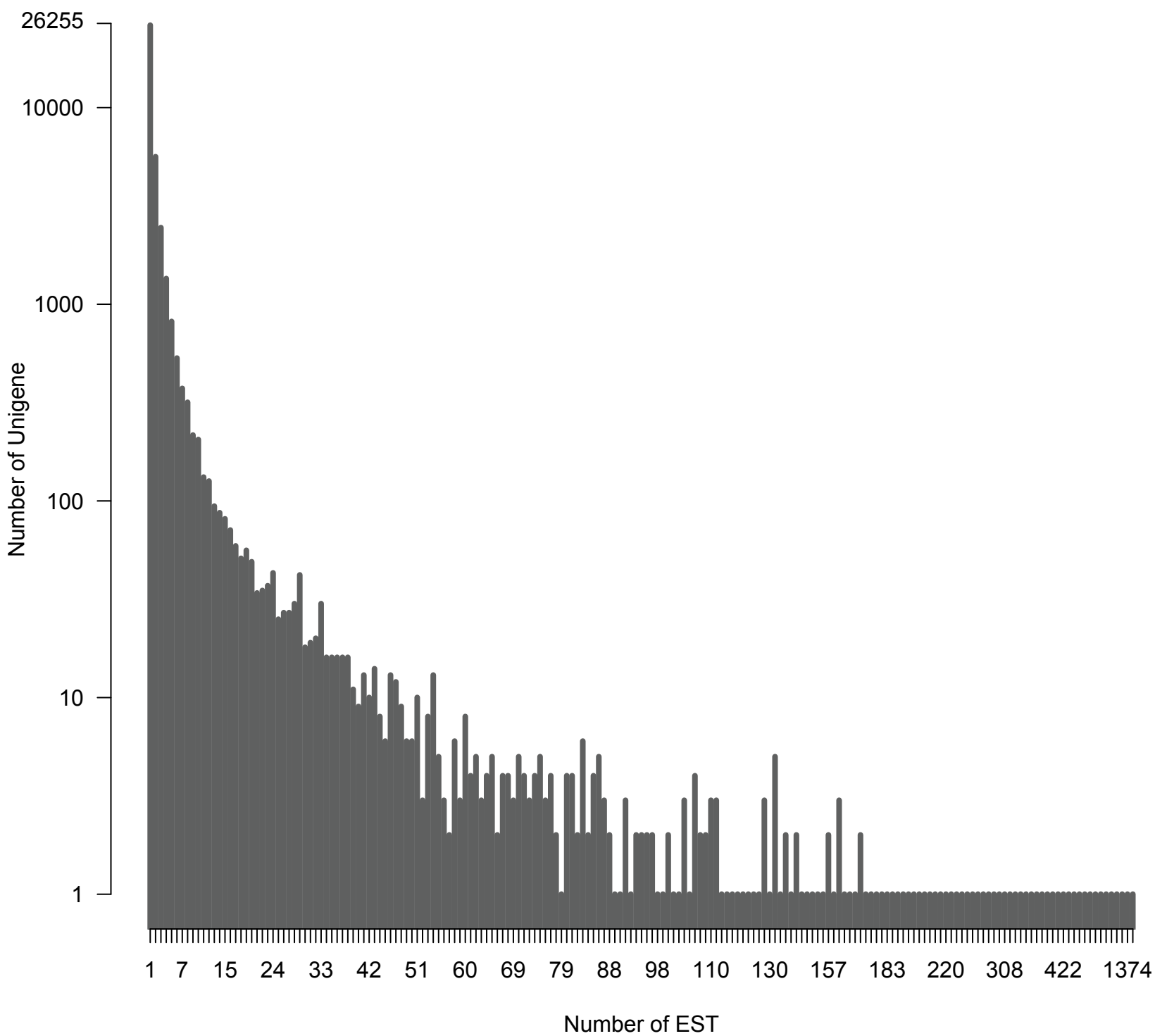

Supplement: Additional file 1 — Distribution of ESTs per unigene. Distribution of EST reads per unigene in the SlF library. The x-axis represents EST reads per unigene and the y-axis the number of unigenes. [file 1471-2164-12-376-S1.PDF]

# Molecular Function

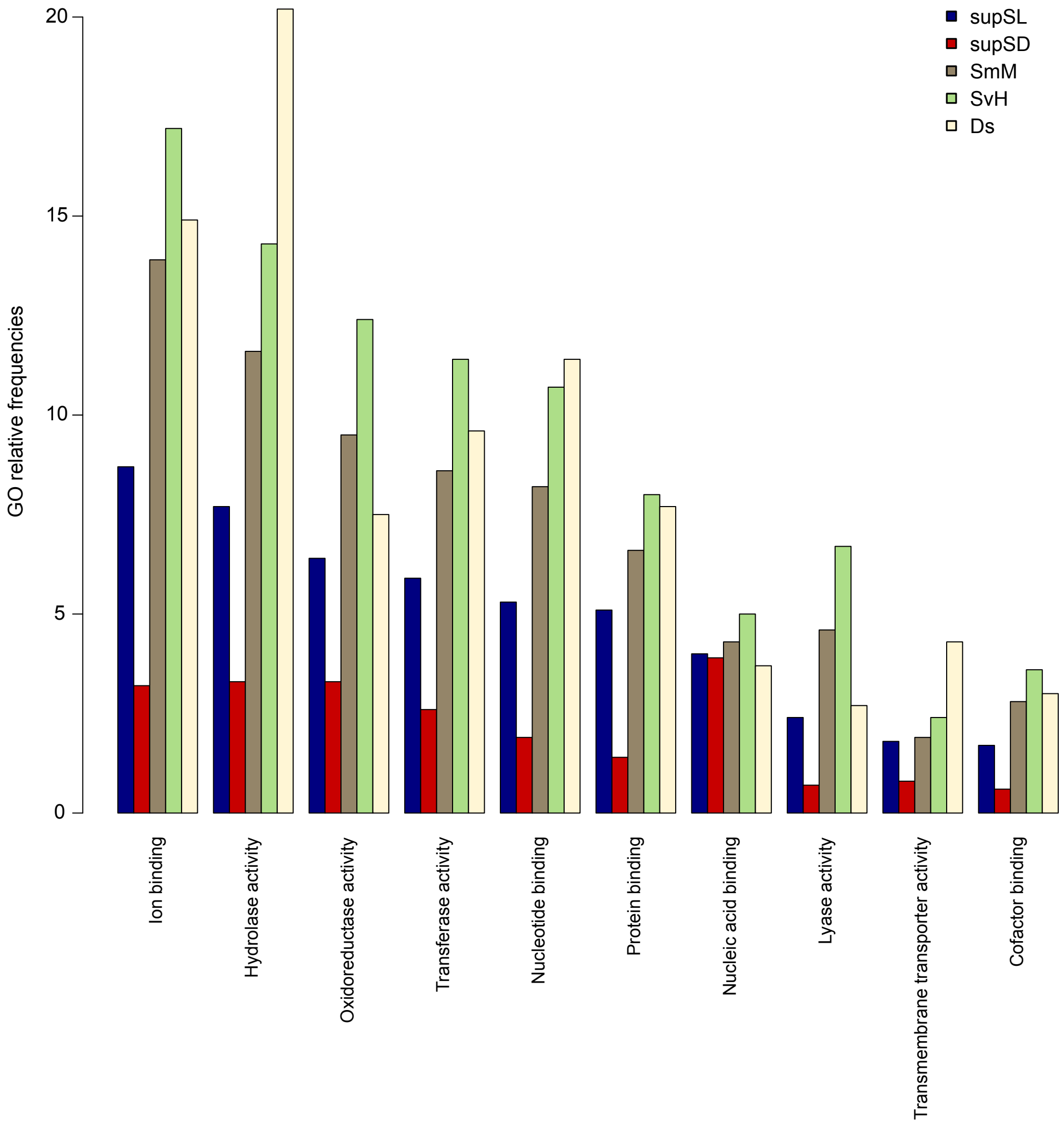

Supplement: Additional file 3 — Relative frequencies of the most represented Molecular Function GO sub-classes across libraries. Additional Figure 2 shows the 10 most frequent molecular function GO terms at level 3 in the five species Silene latifolia, S. dioica, S. marizii, S. vulgaris and Dianthus superbus. [file 1471-2164-12-376-S3.PDF]
